# Killing of Staphylococci by θ-Defensins Involves Membrane Impairment and Activation of Autolytic Enzymes

**DOI:** 10.3390/antibiotics3040617

**Published:** 2014-11-14

**Authors:** Miriam Wilmes, Marina Stockem, Gabriele Bierbaum, Martin Schlag, Friedrich Götz, Dat Q. Tran, Justin B. Schaal, André J. Ouellette, Michael E. Selsted, Hans-Georg Sahl

**Affiliations:** 1 Institute of Medical Microbiology, Immunology and Parasitology, University of Bonn, 53105 Bonn, Germany; E-Mails: S6mastoc@uni-bonn.de (M.S.); g.bierbaum@uni-bonn.de (G.B.); hgsahl@uni-bonn.de (H.-G.S.); 2 Interfaculty Institute of Microbiology and Infection Medicine, Microbial Genetics, University of Tübingen, 72076 Tübingen, Germany; E-Mails: mschlag@frutarom.com (M.S.); friedrich.goetz@uni-tuebingen.de (F.G.); 3 Department of Pathology and Laboratory Medicine, USC Norris Cancer Center, Keck School of Medicine, University of Southern California, Los Angeles, CA 90089-9601, USA; E-Mails: Dat.Tran@med.usc.edu (D.Q.T.); jschaal@usc.edu (J.B.S.); aouellet@med.usc.edu (A.J.O.); selsted@med.usc.edu (M.E.S.)

**Keywords:** antimicrobial peptides, host defense peptides, defensins, antibiotics, mode of action

## Abstract

θ-Defensins are cyclic antimicrobial peptides expressed in leukocytes of Old world monkeys. To get insight into their antibacterial mode of action, we studied the activity of RTDs (rhesus macaque θ-defensins) against staphylococci. We found that in contrast to other defensins, RTDs do not interfere with peptidoglycan biosynthesis, but rather induce bacterial lysis in staphylococci by interaction with the bacterial membrane and/or release of cell wall lytic enzymes. Potassium efflux experiments and membrane potential measurements revealed that the membrane impairment by RTDs strongly depends on the energization of the membrane. In addition, RTD treatment caused the release of Atl-derived cell wall lytic enzymes probably by interaction with membrane-bound lipoteichoic acid. Thus, the premature and uncontrolled activity of these enzymes contributes strongly to the overall killing by θ-defensins. Interestingly, a similar mode of action has been described for Pep5, an antimicrobial peptide of bacterial origin.

## 1. Introduction

Host defense peptides (HDPs) are important effector molecules of the ancient, non-specific innate immune system displaying multiple functions involved in microbial clearance. They may be constitutively expressed or be induced in response to infection or injury, e.g., through activation of Toll-like receptors or pro-inflammatory cytokines. Most peptides exhibit direct antimicrobial activity in the low micromolar concentration range against a large number of microorganisms, including multidrug resistant bacteria [1,2,3]. In higher organisms, HDPs have also been recognized as important immune regulators affecting either stimulation or suppression of immune cell activity [4,5]. Thus, the gene copy number or dysregulated expression of certain HDPs predisposes to various infectious and inflammatory diseases, underlining the importance of these peptides in controlling microbial pathogens [6,7].

Despite the great diversity in their primary structure and amino acid composition, HDPs are typically small (12–50 amino acids), positively charged and able to adopt an amphiphilic structure in solution [1,3,8]. One conserved group of HDPs comprises defensins *sensu stricto*, which were first discovered in mammals and subsequently found in invertebrates, plants and fungi. These peptides are characterized by a disulfide-stabilized β-sheet structure and primarily expressed in epithelial tissues or phagocytic cells [9,10]. Due to structural and functional similarity, it has been proposed that all defensins evolved from a single precursor that can be traced back to prokaryotic origin [11,12].

Mammalian defensins possess potent broad-spectrum activity against both Gram-positive and Gram-negative bacteria, fungi and certain viruses. They are further divided into three groups, α-, β- and θ-defensins, based on their gene structure as well as spacing and pairing of their six conserved cysteine residues. α- And β-defensins are widely expressed in mammals and share a three-stranded antiparallel β-sheet fold, whereas the cyclic θ-defensins have only been isolated from the leukocytes of Old World monkeys [13,14,15]. θ-Defensins arose from a mutated α-defensin gene containing a premature stop codon in its defensin domain. During biogenesis, a nine amino acid segment is excised from the truncated α-defensin precursor and subsequently ligated head to tail to a similar or identical nonapeptide [15].

Rhesus macaques express three θ-defensin precursors, which can pair to generate six different homodimeric and heterodimeric isoforms (rhesus macaque θ-defensins 1 to 6; RTDs). Their concentration in the PMNs differs greatly, with RTD-1 being the most abundant [16]. Interestingly, the cyclic structure seems to be crucial for antimicrobial activity and confers salt resistance up to 150 mM NaCl [14]. In addition, θ-defensins possess potent anti-inflammatory properties *in vitro* and *in vivo* mediated by the suppression of numerous pro-inflammatory cytokines and blockade of TNF-α release [17]. Noteworthy, there are six θ-defensin genes present in the human genome, but a stop codon in the signal sequence blocks their translation. Synthetic or restored products of these pseudogenes termed retrocyclins show remarkable anti-HIV activity by inhibiting virus attachment and entry [18,19,20].

Mode of action studies with defensins from different eukaryotic kingdoms demonstrated that conserved molecules of the microbial cell envelope, which are readily accessible, such as the bacterial cell wall precursor lipid II or fungal sphingolipids, are targets of defensins and an important component of the killing mechanism [21,22,23,24,25,26,27]. Here, we have investigated the mechanism of action of θ-defensin bactericidal activity. Interestingly, RTDs do not interfere with peptidoglycan biosynthesis, but rather induce bacterial lysis in staphylococci by interaction with the bacterial membrane and/or release of autolytic enzymes similar to the lantibiotic Pep5.

## 2. Results and Discussion

**Figure 1:**
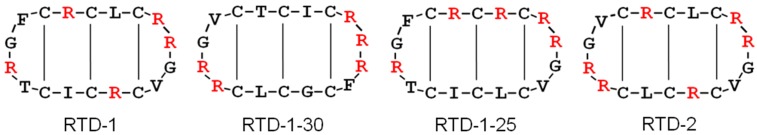
Amino acid sequences of heterodimeric RTD-1 and its two variants and of homodimeric RTD-2. Positively charged residues are marked in red.

The two θ-defensins, RTD-1 and RTD-2 (Figure 1), were initially tested for their activities against different staphylococcal species in a standard broth microdilution assay. Both peptides exhibited potent antimicrobial activity and inhibited growth of the three strains tested at concentrations ranging from 0.5 to 6 μg/mL (Table 1).

**Table 1:** Antimicrobial activity of RTDs against staphylococci in half-concentrated MHB. MIC values (µg/mL) * are expressed as the lowest concentration that caused visible growth inhibition.

	RTD-1	RTD-2	RTD-1-30	RTD-1-25
*S. aureus* SG511-Berlin	6 ± 2	4 ± 0	6 ± 2	8 ± 4
*S. simulans* 22	1.5 ± 0.5	1.5 ± 0.5	3 ± 1	3 ± 1
*S. carnosus* TM300	0.75 ± 0.25	0.5 ± 0	ND	ND

ND, not determined; * Average values obtained from two or more independent experiments (±SD).

### 2.1. Impact on Bacterial Cell Wall Biosynthesis

Fungal [22,25] and invertebrate defensins [24] bind with high affinity to the cell wall building block lipid II, thereby specifically inhibiting peptidoglycan biosynthesis in Gram-positive bacteria. Similarly, lipid II targeting has also been reported for two mammalian defensins, the human α-defensins HNP-1 and β-defensin hBD3, which are both broad-spectrum antimicrobials [21,23].

**Figure 2:**
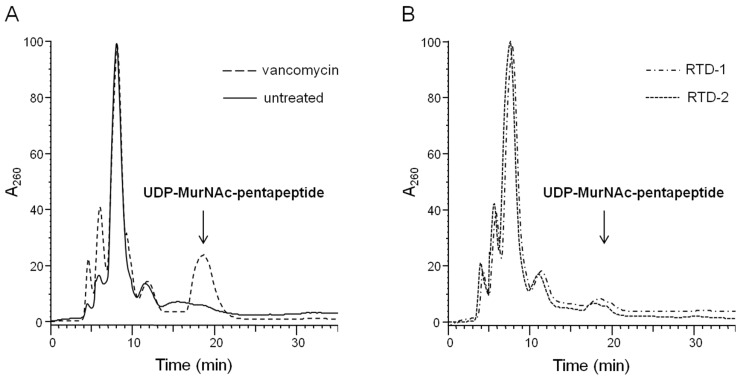
Intracellular accumulation of the final soluble cell wall precursor UDP-MurNAc-pentapeptide in *S. simulans* 22 exposed to θ-defensins. Cells were treated with 10× MIC vancomycin (positive control) (**A**) or RTDs (**B**), incubated for 30 min, and subsequently extracted with boiling water. The cytoplasmic pool of UDP-linked cell wall precursors was analyzed by RP-HPLC.

In order to verify whether θ-defensins also interfere with peptidoglycan biosynthesis, the cytoplasmic level of the cell wall precursor UDP-MurNAc-pentapeptide in *Staphylococcus simulans* 22 treated with RTDs was determined. Accumulation of UDP-MurNAc-pentapeptide is typically induced by antibiotics which inhibit the late, membrane-bound steps of cell wall biosynthesis and has also been demonstrated for hBD3 [23]. As shown in Figure 2, θ-defensins did not cause a significant accumulation of the cell wall precursor compared to vancomycin-treated control cells indicating that the antibiotic action of RTDs differs from that of defensins mentioned above.

### 2.2. Impact on Membrane Integrity

**Figure 3:**
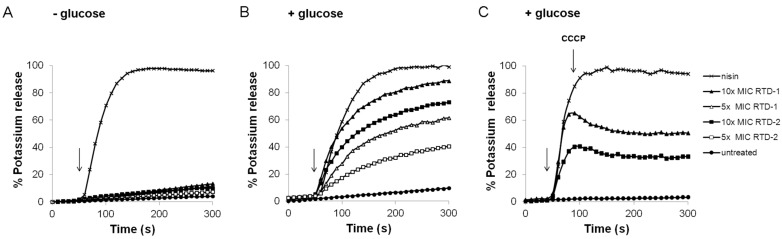
Impact on the membrane integrity of RTD-treated *S. simulans* 22 cells. Potassium efflux was monitored with a potassium-sensitive electrode in absence (**A**) and presence (**B**) of 10 mM glucose. RTD-induced potassium release of energized cells could be blocked by the addition of 5 µM CCCP (carbonyl cyanide m-chlorophenylhydrazone; (**C**). Ion leakage was expressed relative to the total amount of potassium released after addition of 1 µM of the pore-forming lantibiotic nisin (100% efflux). RTDs were added at 5× and 10× MIC; controls were incubated without peptide. The arrows indicate the moment of peptide addition.

Due to their cationic and amphiphilic nature, it is widely believed that the killing activity of HDPs is based on the disruption of the membrane barrier function. To assess the membrane impairment by θ-defensins, the potassium release of whole cells was monitored over a period of 5 min by growing *S. simulans 22* in half-concentrated Mueller-Hinton broth (MHB) and subsequently diluting the cells in choline buffer (see Experimental). Under these conditions, with cells suspended in buffer without an energy source, significant potassium efflux could not be detected in response to RTDs at 5× and 10× MIC (Figure 3A). However, when cells were energized by addition of 10 mM glucose, rapid concentration-dependent ion release occurred after peptide treatment (Figure 3B). In contrast, the activity of the pore-forming lantibiotic nisin—used here as a positive control—was independent of the presence of glucose (Figure 3).

These results suggest that the membrane activity of RTDs depends on the bacterial membrane potential. To investigate this hypothesis further, 5 μM of the ionophore CCCP (carbonyl cyanide m-chlorophenylhydrazone) were added to energized cells shortly after the peptides. CCCP uncouples the proton gradient across the cytoplasmic membrane leading to fast membrane depolarization. Indeed, RTD-induced ion leakage was blocked immediately after CCCP addition (Figure 3C).

Moreover, the membrane potential of *S. simulans* 22 in choline buffer (used for the potassium efflux experiments) was estimated by the distribution of the lipophilic cation TPP^+^ inside and outside the cells. As expected, membrane potential increased by 15–20 mV after incubation with 10 mM glucose (data not shown [28]). Thus, a membrane potential of sufficient magnitude—as builds up after addition of glucose—seems to be essential for the membrane-disrupting activity of RTDs.

Similarly, the membrane potential of *S. aureus* SG511-Berlin was monitored in half-concentrated MHB, which was routinely used for MIC determinations and mode of action studies. In absence of glucose, the peptides did not cause any change of the membrane potential when added at 10× MIC. In contrast, in presence of glucose RTDs caused a reduction of the membrane potential of about 15 mV, which was, however, slowly restored within 20 min of treatment (Supplementary Figure S1).

The requirement for an energized membrane for antibacterial activity has also been described for the cationic antimicrobial peptide Pep5 produced by *S. epidermidis* 5 [29]. Further, Pep5 induces autolysis in *S. simulans* 22 by releasing cell wall lytic enzymes [30,31]. Thus, the question was raised if the membrane impairment alone is sufficient for killing by RTDs or if additional activities are involved in the killing mechanism—such as activation of autolytic enzymes—as described not only for Pep5 [30,31] but also for cationic peptides and proteins in general [32,33].

### 2.3. Impact on Autolytic Enzymes

**Figure 4:**
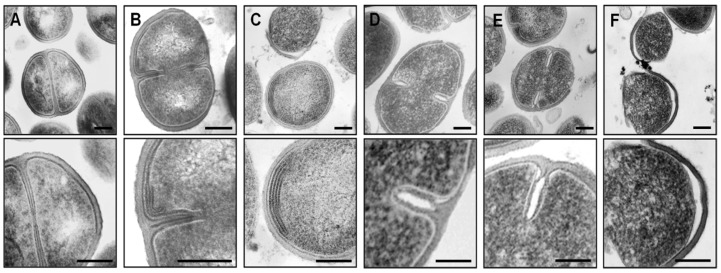
Transmission electron microscopy of *S. aureus* SG511-Berlin treated with 10× MIC RTD-2. (**A**) Untreated control cells. (**B**,**C**) Cells treated for 30 min. Additional membranous structures could be observed. (**D**,**E**,**F**) Cells treated for 60 min. Dividing cells showed degradation of the cell wall in the septum area between two daughter cells (**D**,**E**) or peeling of the cell wall (**F**). Scale bar: 0.2 µm.

RTD-treated cells of *S. aureus* SG511-Berlin grown in half-concentrated MHB were inspected by transmission electron microscopy. After 30 min treatment, additional membranous structures could be observed in many cells (Figure 4A–C), indicating the loss of cytoplasmic content. After 60 min exposure to RTD-2, cells showed evidence of cell wall degradation, particularly in the septum area between two daughter cells (Figure 4D,E). Moreover, in some cells, the cell wall was completely peeled off (Figure 4F). These morphological changes might indicate a premature activation and release of peptidoglycan lytic enzymes (referred to as autolysins) involved in cell separation as has been described for Pep5-treated cells [34]. These results suggest that the release of autolysins, which hydrolyze the glycan chains and peptide bridges of murein, also contributes to the killing activity of RTDs.

To test for release of cell wall hydrolases as a relevant component of the antistaphylococcal activity of RTDs, the supernatant of RTD-treated cells was analyzed for autolytic activity. Hence, *S. aureus* SG511-Berlin was incubated in the presence of RTDs at 10× MIC for 30 or 60 min, harvested and the concentrated supernatants were subjected to SDS-PAGE containing heat-inactivated *M. luteus* cells as substrate. Clear bands indicated the cell wall lytic activity of released enzymes. Cells exposed to Pep5 were included in the study and served as a positive control.

**Figure 5:**
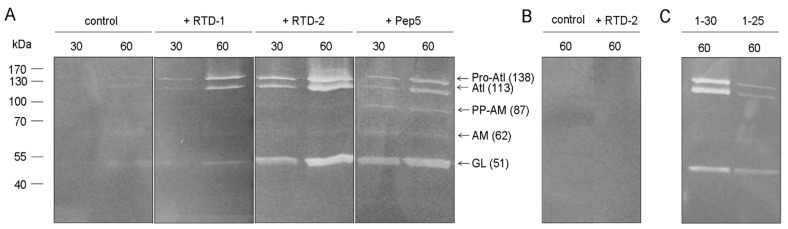
Detection of cell wall lytic enzymes in the supernatant of RTD-treated cells. (**A**) *S. aureus* SG511-Berlin was exposed to 10× MIC of RTDs or Pep5, respectively, for 30 and 60 min. Equal amounts of the concentrated culture supernatant (containing autolysins released from the cell surface) were separated on a 12% SDS-PAGE containing heat-inactivated *M. luteus* cells as substrate. Bands represent cell lysis zones; (**B**) *S. aureus* SA113 *∆atl* treated for 60 min with 10× MIC RTD-2; (**C**) *S. aureus* SG511-Berlin treated for 60 min with two RTD-1 variants (RTD-1-30 and RTD-1-25) which differ in the distribution of positively charged residues. All bands represent differently processed forms of the autolysin Atl as in the *atl* deletion mutant (*∆atl)* the corresponding autolysis bands were missing (**B**). Pro-Atl: Atl with full-length propeptide, Atl: amidase and glucosaminidase, PP-AM: amidase with propeptide, AM: amidase, GL: glucosaminidase.

Autolysins could be observed in all peptide treated samples, whereas hardly any activity was detectable in the untreated control (Figure 5A). Interestingly, all detected bands represent different processed forms of the autolysin Atl since in an *atl* deletion mutant (*S. aureus* SA113 *Δatl*; Figure 5B) corresponding bands were missing after treatment with RTD-2. Atl is a bifunctional autolysin that plays a key role in separating cells after cell division and is highly conserved among staphylococci [35]. Proteolytic processing of the Atl precursor protein of *S. aureus* generates two catalytically active enzymes fused to repeat units, an amidase (AM, 62 kDa; cleaves the amide bond between MurNAc and L-alanine) and a glucosaminidase (GL, 51 kDa; cleaves the β-1,4-glycosidic bond between GlcNAc and adjacent monosaccharides), and both components bind to the septum site of dividing cells [36,37]. In addition to the AM and GL bands, three additional bands with molecular masses of 138 kDa, 113 kDa and 87 kDa could be detected. The 138 kDa band corresponded to the full length protein (Pro-Atl). The 113 kDa and 87 kDa bands presumably represented the unprocessed amidase and glucosaminidase domains after proteolytic cleavage of the signal and propeptide (Atl) and the amidase with the propeptide (PP-AM), respectively (according to Schlag *et al*. [36]).

**Figure 6:**
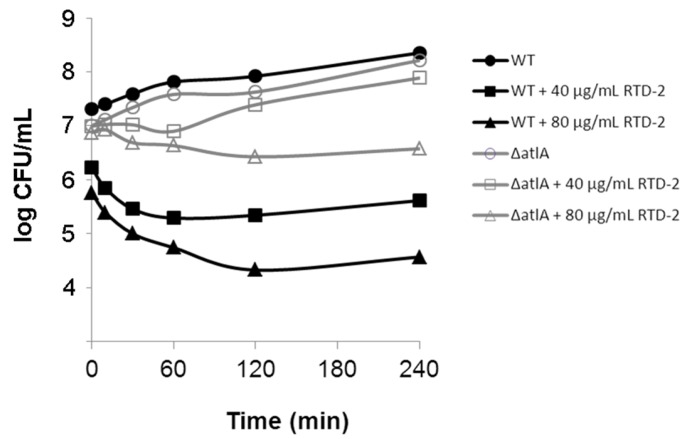
Killing kinetic of *S. aureus* SA113 (WT) and its *atl* deletion mutant (*Δatl*) in presence of 40 and 80 μg/mL RTD-2 (corresponding to 5× and 10× MIC, respectively) over a period of 4 h. The first sample was taken immediately after peptide addition (time point 0). The results given are mean values of at least two independent experiments.

Consistent with the release of the autolysin Atl by RTDs, θ-defensin-mediated killing was diminished in *S. aureus* SA113 *Δatl*. Killing kinetics of the *atl* deletion mutant and its wild-type strain (WT) showed that the mutant was significantly more resistant to the action of RTD-2 (Figure 6). In the course of the experiment, the number of colony forming units (CFU) of the WT was reduced by several log after addition of the peptide, whereas the *atl* deletion mutant was only slightly affected by RTD-2 even at 80 μg/mL (Figure 6).

Recently, it has been reported that the Atl amidase is directed to the septal region by its repeat domains. In this process, *S. aureus* wall teichoic acid (WTA)—present in the old cell wall—acts as a repellent for the repeats, thereby directing the enzymes to the septum, where they bind to and are controlled by lipoteichoic acid (LTA) [36,38]. This suggests that highly cationic molecules such as RTDs and Pep5 bind to the polyanionic LTA, thereby liberating the enzymes such that they can degrade the cell wall in an uncontrolled manner. This view is supported by the fact that only in the WT strain the colony count dropped by 1 to 2 log immediately after RTD addition (data at time point 0, Figure 6). Apparently, the displacement of Atl-derived enzymes from LTA occurs rapidly and is irreversible, such that autolysis can proceed after plating and prevent colony formation. Similar rapid effects described as “contact killing” have been observed with other highly cationic peptides, e.g., hBD3 [39]. How the activity of the Atl enzymes is controlled under coordinated physiological conditions remains to be elucidated.

**Figure 7:**
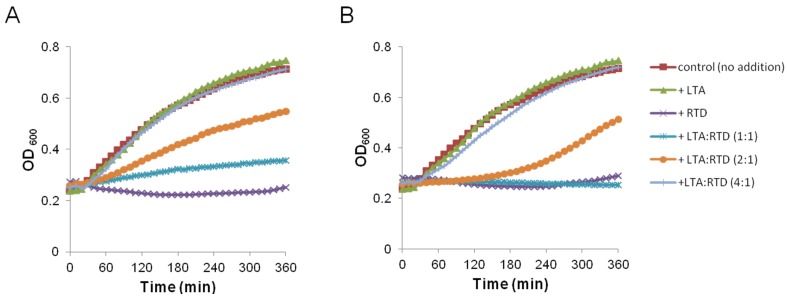
Growth kinetic measurement of *S. aureus* SG511-Berlin in half-concentrated MHB in presence of RTD-1 (**A**) or RTD-2 (**B**) and different molar ratios of LTA. Peptides were added at 10× MIC (corresponding to 28.8 µM and 19.1 µM, respectively).

Consistently, addition of LTA in a 4-fold molar excess in respect to the peptide antagonized the antimicrobial activity of RTDs and resulted in unhindered growth (Figure 7). Moreover, fluorescence microscopy of *S. aureus* SA113 treated with Pep5-Cy3 demonstrated that the peptide localizes preferentially in the septal region of dividing cells (Supplementary Figure S2) similar to Atl repeats [36]. Unfortunately, this experiment could not be performed with RTDs as they do not harbor any free amine group for labeling.

Interestingly, remarkable differences could be revealed between cells treated with RTD-1, its two variants (namely RTD-1-30 and RTD-1-25) and RTD-2 indicating that the number and distribution of positive charges of a given peptide might be of particular relevance for the interaction with LTA and thereby for the release of autolysins. A higher autolysin activity could be detected in the supernatant of cells exposed to RTD-2 (net charge +6) as well as in RTD-1-30 (net charge: +5; Figure 5C) in which the charged residues are clustered on both sides of the molecule (compare Figure 1).

As θ-defensins and Pep5 both interact with membrane-bound lipoteichoic acid, we investigated whether this interaction also facilitates the pore-formation process. For example, the lantibiotic nisin uses lipid II as docking molecule to subsequently form pores in the membrane of susceptible strains [40]. Thus, carboxyfluorescein-loaded liposomes were made of DOPC or DOPC supplemented with 0.5 mol% purified LTA to monitor the efflux of the fluorescent dye after addition of 1 μM of each peptide. However, only minor marker release was observed with pure DOPC vesicles and DOPC doped with LTA indicating that LTA are not involved in the membrane-disrupting activity of RTDs and Pep5 (Supplementary Figure S3).

### 2.4. Activity against Gram-Negative Bacteria

In contrast to Pep5, RTDs also exhibit activity against Gram-negative bacteria over a similar concentration range as against Gram-positives [14,15,41]. Tran *et al*. [41] reported that RTD-1 and RTD-2 effectively permeabilize the outer and inner membrane of *E. coli* ML35-pYC. Consistent with these results, a significant loss of cytoplasmic content could be observed in RTD-2 treated *E. coli* BW25113 cells (Supplementary Figure S4). Moreover, the interaction with the outer membrane seems to differ from hBD3 [42] as blebbing of the outer membrane which is indicative of LPS released from the surface could not be observed (Supplementary Figure S4).

## 3. Experimental Section

### 3.1. Peptide Synthesis and Purification

RTDs were assembled by solid-phase synthesis as described previously [14,15]. Purification of Pep5 and nisin was performed according to the protocol of Sahl and Brandis [43] and Burianek and Yousef [44], respectively.

### 3.2. Determination of Minimal Inhibitory Concentration (MIC)

MIC determinations were carried out in 96-well polypropylene microtiter plates (Nunc^TM^; Thermo Fisher Scientific, Schwerte, Germany) by standard broth microdilution using half-concentrated Mueller-Hinton broth (MHB). Test strains were grown to an optical density at 600 nm (OD_600_) of 1 and subsequently diluted to 1–2 × 10^5^ cells/mL. Then, 50 µL of the bacterial suspension were mixed with 50 µL of the peptide solution. MICs were read after 24 h of incubation at 37 °C without agitation. The results given are mean values of at least two independent experiments performed in duplicate.

### 3.3. Bacterial Killing Kinetics 

Cells were grown in half-concentrated MHB to an OD_600_ of 0.1. Defensins were added in concentrations corresponding to 5× or 10× MIC (as determined after 24 h). At defined time intervals, 40 μL aliquots of the culture were taken, diluted in 360 μL 10 mM potassium phosphate buffer (pH 7) and 100 μL of appropriate dilutions were plated in triplicate on Mueller-Hinton II agar plates (Becton Dickinson GmbH). The plates were incubated overnight at 37 °C and the number of colony forming units (CFU) was calculated based on the respective dilution factor. An untreated culture was run as a control. The results given are mean values of at least two independent experiments.

### 3.4. Intracellular Accumulation of the Final Soluble Cell Wall Precursor UDP-MurNAc-Pentapeptide

Antibiotics that interfere with the late, membrane-bound steps of peptidoglycan biosynthesis, trigger the accumulation of UDP-MurNAc-pentapeptide in the cytoplasm which can be isolated and detected by HPLC. Analysis of the cytoplasmic peptidoglycan precursor pool was performed as described previously [23,24]. Briefly, *S. simulans* 22 was grown to an OD_600_ of 0.5 in half-concentrated MHB and supplemented with 130 μg/mL of chloramphenicol. After 15 min of incubation, defensins or vancomycin, respectively, were added at 10× MIC and the samples were further incubated for 30 min. Then, cells were harvested and treated with two volumes boiling water for 15 min. Insoluble components were removed by centrifugation and the supernatants analyzed by reversed-phase high pressure liquid chromatography (RP-HPLC) in 50 mM sodium phosphate buffer (pH 5.2) under isocratic conditions on a Nucleosil 100-C18 column (Schambeck SFD GmbH, Bad Honnef, Germany).

### 3.5. Potassium Release from Whole Cells

Potassium efflux from whole cells was monitored with a MI-442 potassium electrode and a MI-409F reference electrode (Microelectrodes Inc., Bedford, MA, USA) connected to a microprocessor pH meter (pH 213; HANNA^®^ Instruments, Kehl, Germany).

*S. simulans* 22 was grown in half-concentrated MHB (±10 mM glucose) at 37 °C to an OD_600_ of 1 to 1.5. Then, cells were harvested by centrifugation (4000 rpm, 3 min, 4 °C), washed with prechilled choline buffer (300 mM choline chloride, 30 mM MES, 20 mM Tris; pH 6.5) and resuspended in the same buffer (±10 mM glucose) to a final OD_600_ of 30. For each measurement, cells were diluted in choline buffer (±10 mM glucose) to an OD_600_ of 3, and the potassium release was monitored for 5 min at room temperature. Peptides were added at 5× and 10× MIC. Potassium concentrations were calculated from the measured voltage according to Orlov *et al*. [45] and expressed relative to the total amount of potassium released after addition of 1 µM of the pore-forming lantibiotic nisin (100% efflux). Results given are mean values of three independent experiments.

### 3.6. Estimation of Membrane Potential

Cells were grown in half-concentrated MHB (±10 mM glucose) to an OD_600_ of 0.5 to 0.6. To monitor the membrane potential, 1 μCi/mL of [^3^H]tetraphenylphosphonium bromide (TPP^+^; 26 Ci/mM; Hartmann Analytic GmbH, Braunschweig, Germany) was added (the lipophilic TPP^+^ diffuses across the bacterial membrane in response to a trans-negative membrane potential). The culture was treated with defensins at 10× MIC, sample aliquots of 100 μL were filtered through cellulose acetate filters (pore size 0.2 μm; Whatman^TM^, Dassel, Germany) and washed twice with 5 mL of 50 mM potassium phosphate buffer (pH 7). The filters were dried, placed into 5 mL scintillation fluid and the radioactivity was measured with a liquid scintillation counter for 5 min per filter. Non specific TPP^+^ binding was determined by measuring the TPP^+^ incorporation into cells treated with 10% butanol (v/v); the total radioactivity was measured using unfiltered 100 μL sample aliquots. The pore-forming lantibiotic nisin was used as a control. For calculation of the membrane potential (∆ψ), the TPP^+^ concentrations were applied into the Nernst equation ∆ψ = (2.3 × *R* × *T/F*) × log [(TPP^+^ inside)/(TPP^+^ outside)], where *T* is the absolute temperature, *R* is the universal gas constant and *F* is the Faraday constant. A mean ∆ψ was calculated from at least two independent experiments.

### 3.7. Zymogram Analysis

Cell wall lytic enzymes in the supernatant of RTD- and Pep5-treated cells were analyzed by zymograms. For this, *S. aureus* SG511-Berlin was grown in half-concentrated MHB to an OD_600_ of 0.6. Then, cells were harvested by centrifugation (4000 rpm, 5 min, 4 °C) and washed with 10 mM sodium phosphate buffer (SPB; pH 7.4). Finally, cells were resuspended in 10% MHB (in 10 mM SPB; pH 7.4) and aliquots of 2.5 mL were incubated with peptides corresponding to 10× MIC. After incubation for 30 or 60 min, cells were pelleted (10,000 rpm, 5 min, 4 °C). The supernatant containing released proteins was concentrated to a volume of 50 µL using VivaSpin-columns (Sartorius AG, Göttingen, Germany) according to the manufacturer’s instructions. Equal amounts of the enzyme extract were loaded onto a polyacrylamide gel containing heat-killed *M. luteus* DSM 1790 cells as substrate. After the run, the gel was washed three times with distilled water for 15 min before overnight incubation in buffer (50 mM Tris-HCl, pH 7.5; 10 mM CaCl_2_, 10 mM MgCl_2_, 0.1% Triton X-100, v/v) at 37 °C. Lytic activity was observed as clear zones against an opaque background. To gain a higher contrast, gels were stained with 0.1% methylene blue (w/v) for 15 min and washed with distilled water until clear bands became visible.

### 3.8. Transmission Electron Microscopy

Cells were grown in half-concentrated MHB to an OD_600_ of 0.6. Aliquots of 5 mL were exposed to RTDs (at 10× MIC) for 30 min or 60 min at 37 °C. Afterwards, the bacteria were harvested by centrifugation (5000 rpm, 5 min, 4 °C), resuspended in 0.1 M SPB (pH 7.4) containing 3% glutaraldehyde (v/v) and fixed overnight at 4 °C. After washing the cells three times for 10 min with 0.1 M SPB (pH 7.4), they were postfixed in 2% phosphate-buffered osmium tetroxide (v/v) at 4 °C for 2 h. Subsequently, the samples were dehydrated with increasing concentrations of ethanol beginning with 30%. The dehydrated cells were incubated three times in propylene oxide for 5 min, followed by a treatment with a 1:1 mixture of polypropylene oxide and Epon-812 (v/v; Science Services, München, Germany) overnight at RT. Finally, cells were embedded in Epon-812 and incubated for polymerization at 60 °C for 48 h. Thin sections (60 nm) were contrasted with 3% uranyl acetate and 0.3% lead citrate and subsequently examined with an EM900 electron microscope (Zeiss; Oberkochen, Germany) at 50 kV.

### 3.9. Growth Kinetic in Presence of LTA

LTA isolated from *S. aureus* (Sigma-Aldrich, Taufkirchen, Germany) was tested for antagonization of antimicrobial activity. Therefore, *S. aureus* SG511-Berlin was grown in half-concentrated MHB to an OD_600_ of 0.5 to 0.6. Then, 100 µL of the cell suspension were added to 100 µL of RTD-1 or RTD-2 (at 10× MIC) preincubated with LTA in a molar ratios of 1:1, 1:2 and 1:4 in respect to the peptide. Cell growth was measured on a microplate reader (Sunrise^TM^; Tecan, Crailsheim, Germany) over a period of 6 h at 37 °C and obtained data were analyzed by Magellan^TM^ data analysis software [46]. Results given are mean values of at least two independent experiments performed in triplicate.

## 4. Conclusions

Here we report that RTDs do not only impair the membrane barrier function, but also liberate the staphylococcal autolysin Atl from LTA in a similar way as the lantibiotic Pep5. These peptides share some features such as the regular spacing of positively charged residues and a structure stabilized by disulfide bridges or lanthionines, respectively. Thus, the antimicrobial activity may rather depend on the overall conformation and charge distribution than on the primary sequence. However, Pep5 and RTDs differ in their activity spectrum, in particular in their efficacy against Gram-negative bacteria. This demonstrates that the physico-chemical properties which provide the basis for the activity against Gram-positive bacteria are not relevant for the killing of Gram-negative bacteria where—in case of RTDs—membrane permeabilization may be the dominant killing activity.

## Supplementary Files

Supplementary File 1

## References

[1] Jenssen H., Hamill P., Hancock R.E. (2006). Peptide antimicrobial agents. Clin. Microbiol. Rev..

[2] Yeung A.T., Gellatly S.L., Hancock R.E. (2011). Multifunctional cationic host defence peptides and their clinical applications. Cell. Mol. Life Sci..

[3] Zasloff M. (2002). Antimicrobial peptides of multicellular organisms. Nature.

[4] Hilchie A.L., Wuerth K., Hancock R.E. (2013). Immune modulation by multifaceted cationic host defense (antimicrobial) peptides. Nat. Chem. Biol..

[5] Selsted M.E., Ouellette A.J. (2005). Mammalian defensins in the antimicrobial immune response. Nat. Immunol..

[6] Ganz T., Metcalf J.A., Gallin J.I., Boxer L.A., Lehrer R.I. (1988). Microbicidal/cytotoxic proteins of neutrophils are deficient in two disorders: Chediak-Higashi syndrome and “specific” granule deficiency. J. Clin. Invest..

[7] Wehkamp J., Salzman N.H., Porter E., Nuding S., Weichenthal M., Petras R.E., Shen B., Schaeffeler E., Schwab M., Linzmeier R. (2005). Reduced Paneth cell alpha-defensins in ileal Crohn’s disease. Proc. Natl. Acad. Sci. USA.

[8] Ganz T., Lehrer R.I. (1998). Antimicrobial peptides of vertebrates. Curr. Opin. Immunol..

[9] Ganz T. (2003). Defensins: Antimicrobial peptides of innate immunity. Nat. Rev. Immunol..

[10] Hancock R.E., Diamond G. (2000). The role of cationic antimicrobial peptides in innate host defences. Trends Microbiol..

[11] Gao B., Rodriguez Mdel C., Lanz-Mendoza H., Zhu S. (2009). AdDLP, a bacterial defensin-like peptide, exhibits anti-Plasmodium activity. Biochem. Biophys. Res. Commun..

[12] Yeaman M.R., Yount N.Y. (2007). Unifying themes in host defence effector polypeptides. Nat. Rev. Microbiol..

[13] Garcia A.E., Osapay G., Tran P.A., Yuan J., Selsted M.E. (2008). Isolation, synthesis, and antimicrobial activities of naturally occurring theta-defensin isoforms from baboon leukocytes. Infect. Immun..

[14] Tang Y.Q., Yuan J., Osapay G., Osapay K., Tran D., Miller C.J., Ouellette A.J., Selsted M.E. (1999). A cyclic antimicrobial peptide produced in primate leukocytes by the ligation of two truncated alpha-defensins. Science.

[15] Tran D., Tran P.A., Tang Y.Q., Yuan J., Cole T., Selsted M.E. (2002). Homodimeric theta-defensins from rhesus macaque leukocytes: isolation, synthesis, antimicrobial activities, and bacterial binding properties of the cyclic peptides. J. Biol. Chem..

[16] Tongaonkar P., Tran P., Roberts K., Schaal J., Osapay G., Tran D., Ouellette A.J., Selsted M.E. (2011). Rhesus macaque theta-defensin isoforms: expression, antimicrobial activities, and demonstration of a prominent role in neutrophil granule microbicidal activities. J. Leukoc. Biol..

[17] Schaal J.B., Tran D., Tran P., Osapay G., Trinh K., Roberts K.D., Brasky K.M., Tongaonkar P., Ouellette A.J., Selsted M.E. (2012). Rhesus macaque theta defensins suppress inflammatory cytokines and enhance survival in mouse models of bacteremic sepsis. PLoS One.

[18] Cole A.M., Hong T., Boo L.M., Nguyen T., Zhao C., Bristol G., Zack J.A., Waring A.J., Yang O.O., Lehrer R.I. (2002). Retrocyclin: A primate peptide that protects cells from infection by T- and M-tropic strains of HIV-1. Proc. Natl. Acad. Sci. USA.

[19] Gallo S.A., Wang W., Rawat S.S., Jung G., Waring A.J., Cole A.M., Lu H., Yan X., Daly N.L., Craik D.J. (2006). Theta-defensins prevent HIV-1 Env-mediated fusion by binding gp41 and blocking 6-helix bundle formation. J. Biol. Chem..

[20] Venkataraman N., Cole A.L., Ruchala P., Waring A.J., Lehrer R.I., Stuchlik O., Pohl J., Cole A.M. (2009). Reawakening retrocyclins: ancestral human defensins active against HIV-1. PLoS Biol..

[21] De Leeuw E., Li C., Zeng P., Diepeveen-de Buin M., Lu W.Y., Breukink E., Lu W. (2010). Functional interaction of human neutrophil peptide-1 with the cell wall precursor lipid II. FEBS Lett..

[22] Oeemig J.S., Lynggaard C., Knudsen D.H., Hansen F.T., Norgaard K.D., Schneider T., Vad B.S., Sandvang D.H., Nielsen L.A., Neve S. (2012). Eurocin, a new fungal defensin: Structure, lipid binding, and its mode of action. J. Biol. Chem..

[23] Sass V., Schneider T., Wilmes M., Korner C., Tossi A., Novikova N., Shamova O., Sahl H.G. (2010). Human beta-defensin 3 inhibits cell wall biosynthesis in Staphylococci. Infect. Immun..

[24] Schmitt P., Wilmes M., Pugniere M., Aumelas A., Bachere E., Sahl H.G., Schneider T., Destoumieux-Garzon D. (2010). Insight into invertebrate defensin mechanism of action: Oyster defensins inhibit peptidoglycan biosynthesis by binding to lipid II. J. Biol. Chem..

[25] Schneider T., Kruse T., Wimmer R., Wiedemann I., Sass V., Pag U., Jansen A., Nielsen A.K., Mygind P.H., Raventos D.S. (2010). Plectasin, a fungal defensin, targets the bacterial cell wall precursor Lipid II. Science.

[26] Thevissen K., Warnecke D.C., Francois I.E., Leipelt M., Heinz E., Ott C., Zahringer U., Thomma B.P., Ferket K.K., Cammue B.P. (2004). Defensins from insects and plants interact with fungal glucosylceramides. J. Biol. Chem..

[27] Thevissen K., de Mello Tavares P., Xu D., Blankenship J., Vandenbosch D., Idkowiak-Baldys J., Govaert G., Bink A., Rozental S., de Groot P.W. (2012). The plant defensin RsAFP2 induces cell wall stress, septin mislocalization and accumulation of ceramides in *Candida albicans*. Mol. Microbiol..

[28] Wilmes M., Sahl H.G. (2012).

[29] Sahl H.G. (1985). Influence of the staphylococcinlike peptide Pep 5 on membrane potential of bacterial cells and cytoplasmic membrane vesicles. J. Bacteriol..

[30] Bierbaum G., Sahl H.G. (1985). Induction of autolysis of staphylococci by the basic peptide antibiotics Pep 5 and nisin and their influence on the activity of autolytic enzymes. Arch. Microbiol..

[31] Bierbaum G., Sahl H.G. (1987). Autolytic system of *Staphylococcus simulans* 22: Influence of cationic peptides on activity of N-acetylmuramoyl-L-alanine amidase. J. Bacteriol..

[32] Lahav M., Ginsburg I. (1977). Effect of leukocyte hydrolases on bacteria. X. The role played by leukocyte factors, cationic polyelectrolytes, and by membrane-damaging agents in the lysis of *Staphylococcus aureus*: Relation to chronic inflammatory processes. Inflammation.

[33] Ginsburg I., Lahav M. (1983). Lysis and biodegradation of microorganisms in infectious sites may involve cooperation between leukocyte, serum factors and bacterial wall autolysins: A working hypothesis. Eur. J. Clin. Microbiol..

[34] Bierbaum G., Sahl H.G., Jung G., Sahl H.G. (1991). Induction of autolysis of *Staphylococcus simulans* 22 by Pep5 and nisin and influence of the cationic peptides on the activity of the autolytic enzymes. Nisin and Novel Lantibiotics.

[35] Albrecht T., Raue S., Rosenstein R., Nieselt K., Götz F. (2012). Phylogeny of the staphylococcal major autolysin and its use in genus and species typing. J. Bacteriol..

[36] Schlag M., Biswas R., Krismer B., Kohler T., Zoll S., Yu W., Schwarz H., Peschel A., Götz F. (2010). Role of staphylococcal wall teichoic acid in targeting the major autolysin Atl. Mol. Microbiol..

[37] Yamada S., Sugai M., Komatsuzawa H., Nakashima S., Oshida T., Matsumoto A., Suginaka H. (1996). An autolysin ring associated with cell separation of *Staphylococcus aureus*. J. Bacteriol..

[38] Zoll S., Schlag M., Shkumatov A.V., Rautenberg M., Svergun D.I., Götz F., Stehle T. (2012). Ligand-binding properties and conformational dynamics of autolysin repeat domains in staphylococcal cell wall recognition. J. Bacteriol..

[39] Sass V., Pag U., Tossi A., Bierbaum G., Sahl H.G. (2008). Mode of action of human β-defensin 3 against *Staphylococcus aureus* and transcriptional analysis of responses to defensin challenge. Int. J. Med. Microbiol..

[40] Wiedemann I., Breukink E., van Kraaij C., Kuipers O.P., Bierbaum G., de Kruijff B., Sahl H.G. (2001). Specific binding of nisin to the peptidoglycan precursor lipid II combines pore formation and inhibition of cell wall biosynthesis for potent antibiotic activity. J. Biol. Chem..

[41] Tran D., Tran P.A., Tang Y.Q., Yuan J., Cole T., Selsted M.E. (2008). Microbicidal properties and cytocidal selectivity of rhesus macaque theta defensins. Antimicrob. Agents Chemother..

[42] Wilmes M., Sahl H.G. (2014). Defensin-based anti-infective strategies. Int. J. Med. Microbiol..

[43] Sahl H.G., Brandis H. (1981). Production, purification and chemical properties of an antistaphylococcal agent produced by *Staphylococcus epidermidis.*. J. Gen. Microbiol..

[44] Burianek L.L., Yousef A.E. (2000). Solvent extraction of bacteriocins from liquid cultures. Lett. Appl. Microbiol..

[45] Orlov D.S., Nguyen T., Lehrer R.I. (2002). Potassium release, a useful tool for studying antimicrobial peptides. J. Microbiol. Methods.

[46] (2010). Magellan^TM^ Software V7.0.

