# Supplementary material for: Killing of Staphylococci by θ-Defensins Involves Membrane Impairment and Activation of Autolytic Enzymes
Source: Antibiotics (Basel). 2014 Nov 14;3(4):617–31. doi: 10.3390/antibiotics3040617 (PMC4306331; doi:10.3390/antibiotics3040617)
Supplement: Supplementary File 1 [file antibiotics-03-00617-s001.pdf]

Supplementary Material

Supplementary Figure S1. Membrane potential of *S. aureus* SG511-Berlin in half-concentrated MHB in absence (**A**) and presence (**B**) of 10 mM glucose. RTDs were added in concentrations corresponding to 10 × MIC. The pore-forming peptide nisin was used as a control. Arrows indicate the moment of peptide addition.

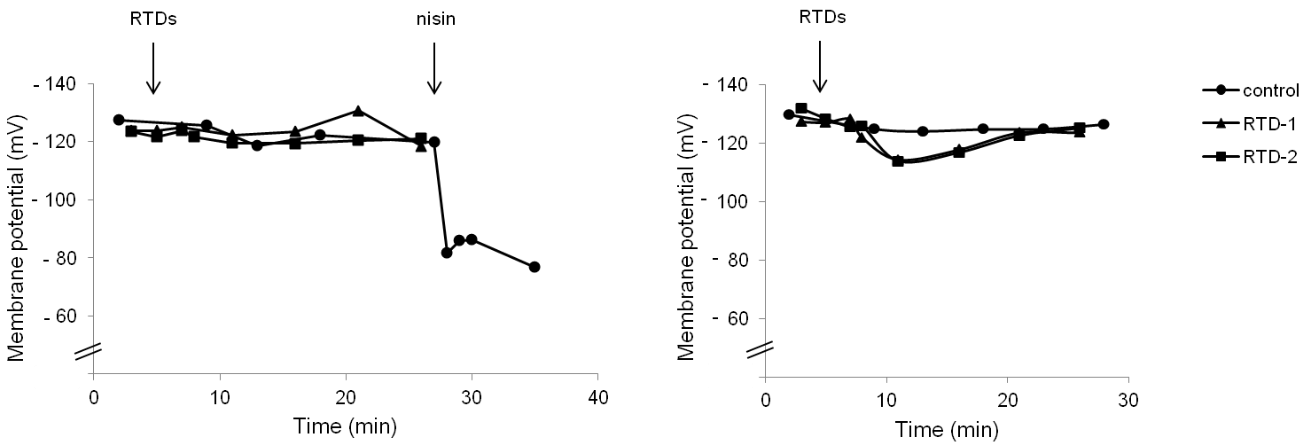

Supplementary Figure S2. Localization of Pep5-Cy3 in *S. aureus* SA113. Cells were grown to exponential phase, incubated with Pep5-Cy3 for 5 min and inspected by fluorescence microscopy.

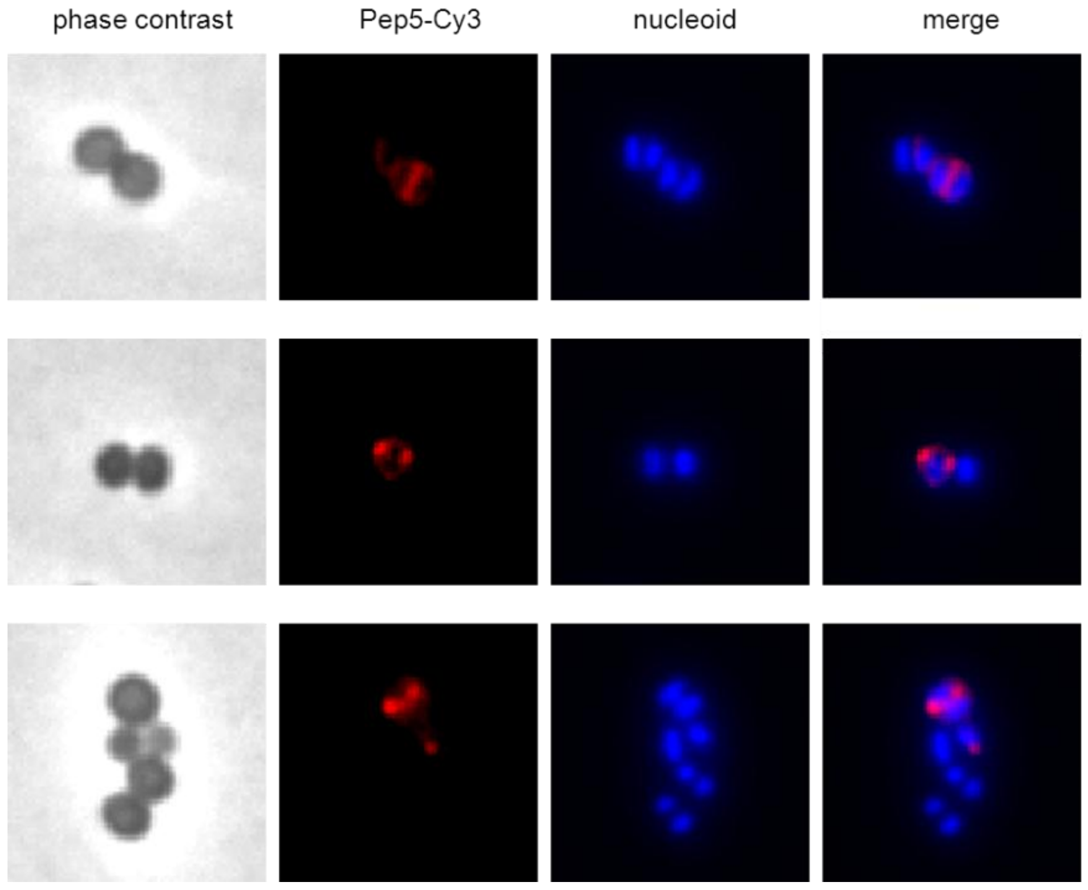

Supplementary Figure S3. Carboxyfluorescein (CF) release from liposomes made of DOPC and 0.5 mol% LTA. RTDs (**A**) and Pep5 (**B**) were added at 1 µM. Marker release was expressed relative to the amount of CF released after addition of Triton X-100 (100% efflux). Pure DOPC vesicles were used as a control.

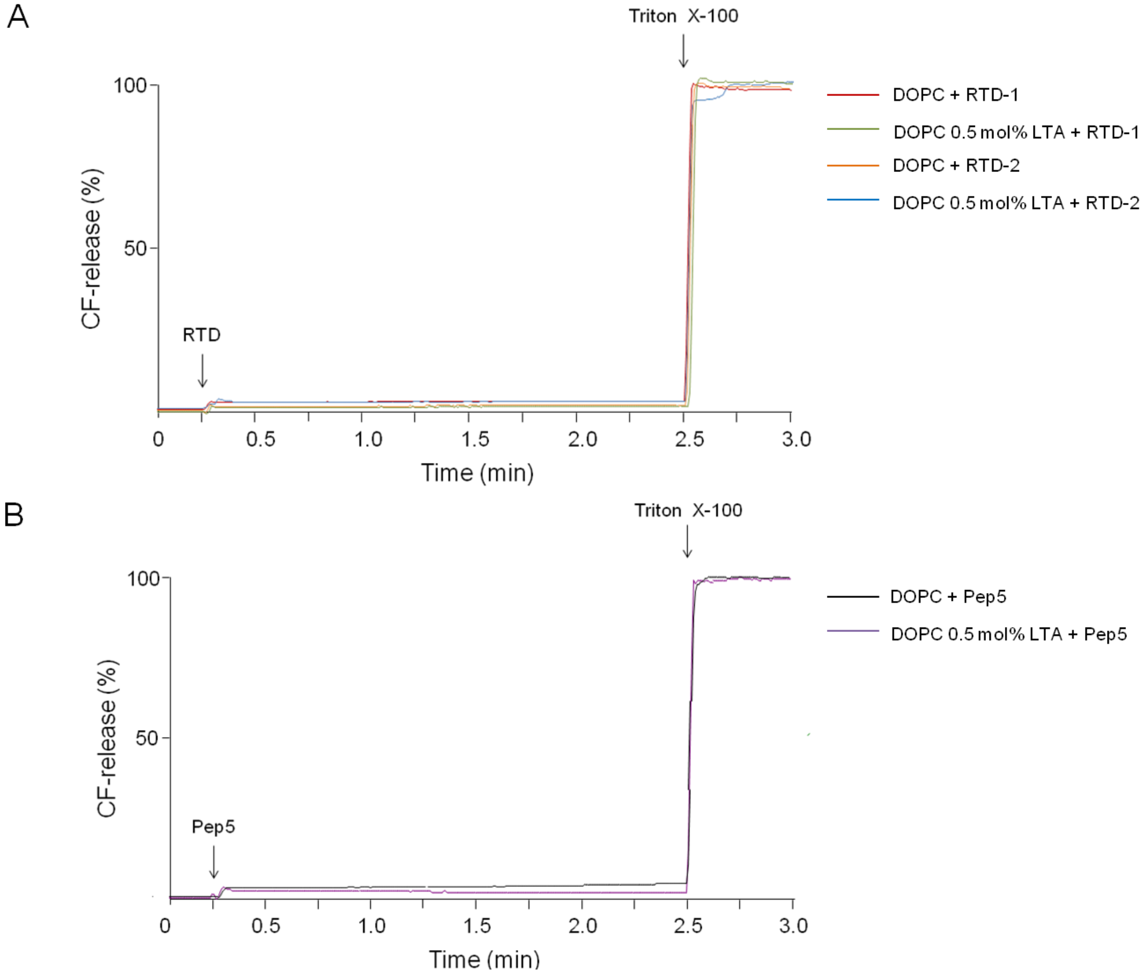

Supplementary Figure S4. Transmission electron microscopy of *E. coli* BW25113 treated with 10× MIC RTD-2. (**A**) Untreated control cells; (**B**) Cells treated for 15 min; (**C**) Cells treated for 30 min. Scale bar: 0.5 µm.

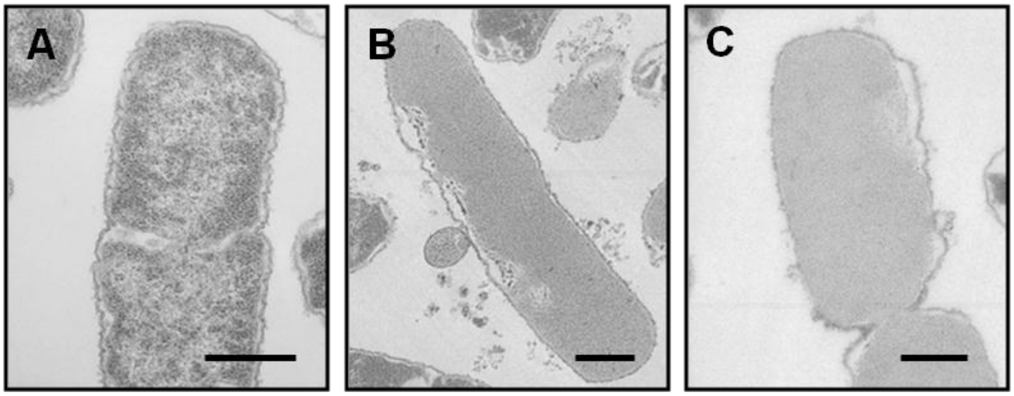

Supplementary Experimental Methods

Supplementary Table S1. Strains used in this study.

Strain	Relevant characteristic(s)	Reference/Source
S. aureus SG511-Berlin	Mutation in gene *graS*	[1]
S. aureus SA113	Derivative of *S. aureus* NCTC 8325	[2]
S. aureus SA113 Δ*atl*	*atlA* deletion mutant of strain SA113 (Δ*atlA*::spc)	[3]
S. simulans 22	Indicator strain	[4]
S. carnosus TM300	Indicator strain	[5]
E. coli BW25113	K-12 strain	[6]

CF Efflux

Vesicles were made of pure DOPC (1,2-dioleoyl-sn-glycero-3-phosphocholine; Avanti polar lipids, Alabaster, AL, USA) or DOPC supplemented with 0.5 mol% LTA (Sigma-Aldrich, Taufkirchen, Germany) referring to the total amount of phospholipids. For this, 4 µM DOPC were mixed with LTA and the solvent was evaporated under a nitrogen stream. Then, the lipids were resuspended in 600 µL buffer (50 mM MES-KOH; 100 mM K_2SO_4; pH 6 or 10 mM Tris-HCl; 0.85% NaCl; pH 7.2) containing 50 mM carboxyfluorescein (CF). Unilamellar vesicles were prepared by the extrusion technique (polycarbonate filter, pore size 0.4 µm; Whatman™, Dassel, Germany). Liposomes were separated from unencapsulated CF by gel filtration using sephadex G-50 (Sigma-Aldrich).

The CF-loaded vesicles were diluted in 1.5 mL buffer (see above) at a final concentration of 25 µM phospholipid on a phosphorous base. The fluorescence intensity was measured at 520 nm (excitation at 492 nm) on a RF-5301 spectrophotometer (Shimadzu, Duisburg, Germany) for 3 min at RT. Peptides were added after 25 s at concentrations of 1 µM. To determine 100% marker release, 20 µL of 20% Triton X-100 (v/v) were added at the end of each measurement.

Fluorescence Microscopy

Pep5 was fluorescently labeled using the Cy3 Mono Reactive Dye Pack (GE Healthcar, Buckinghamshire, UK) according to the manufacturer's instructions.

For localization of Pep5, *S. aureus* SA113 was grown to mid-exponential phase, incubated with Pep5-Cy3 and DAPI (4',6-Diamidin-2-phenylindol; 0.25 µg/mL) for 5 min at 37 °C and subsequently washed three times with SPB (10 mM, pH 7.5). Then, 10 µl of cell suspension were applied on glass slides covered with 1% agarose and examined with a DMRB fluorescence microscope (Leica, Wetzlar, Germany).

References

1. Sass, P.; Bierbaum, G. Native graS mutation supports the susceptibility of *Staphylococcus aureus* strain SG511 to antimicrobial peptides. *Int. J. Med. Microbiol.* **2009**, *299*, 313–322.
2. Iordanescu, S.; Surdeanu, M. Two restriction and modification systems in *Staphylococcus aureus* NCTC8325. *J. Gen. Microbiol.* **1976**, *96*, 277–281.
3. Biswas, R.; Voggu, L.; Simon, U.K.; Hentschel, P.; Thumm, G.; Gotz, F. Activity of the major staphylococcal autolysin Atl. *FEMS Microbiol. Lett.* **2006**, *259*, 260–268.
4. Sahl, H.G.; Brandis, H. Production, purification and chemical properties of an antistaphylococcal agent produced by *Staphylococcus epidermidis*. *J. Gen. Microbiol.* **1981**, *127*, 377–384.
5. Schleifer, K.H.; Fischer, U. Description of a new species of the genus Staphylococcus: *Staphylococcus carnosu*s. *Int. J. System. Bacteriol.* **1983**, *32*, 153–156.
6. Datsenko, K.A.; Wanner, B.L. One-step inactivation of chromosomal genes in *Escherichia coli* K-12 using PCR products. *Proc. Natl. Acad. Sci. USA* **2000**, *97*, 6640–6645.

© 2014 by the authors; licensee MDPI, Basel, Switzerland. This article is an open access article distributed under the terms and conditions of the Creative Commons Attribution license (http://creativecommons.org/licenses/by/4.0/).
